# Stronger Associations Between Sleep and Mental Health in Adults with Autism: A UK Biobank Study

**DOI:** 10.1007/s10803-021-05382-1

**Published:** 2021-12-03

**Authors:** Lisa M. Henderson, M. St Clair, V. Knowland, E. van Rijn, S. Walker, M. G. Gaskell

**Affiliations:** 1 Department of Psychology, University of York, York, YO10 5DD UK; 2 Department of Psychology, University of Bath, Bath, UK; 3 Department of Speech and Language Sciences, University of Newcastle, Newcastle, Australia

**Keywords:** Accelerometer, Actigraphy, Mental health, UK Biobank, Autism, Sleep

## Abstract

This study examined sleep and its cognitive and affective correlates in adults with and without autism spectrum disorder (ASD), utilizing UK Biobank data. There were no group differences in subjective sleep duration [n = 220 ASD; n = 2200 general population (GP)]. Accelerometer measures of sleep duration or nighttime activity did not differ by group, but sleep efficiency was marginally lower in ASD (n = 83 ASD; n = 824 GP). Sleep efficiency was associated with wellbeing and mental health, and pathways between accelerometer sleep measures and wellbeing and mental health were significantly stronger for adults with ASD (who also reported substantially poorer wellbeing and > 5 × likelihood of experiencing mental distress). These findings highlight the need to monitor sleep to maintain good mental health in adult ASD.

**Supplementary Information:**

The online version contains supplementary material available at 10.1007/s10803-021-05382-1.

## Introduction

Autism spectrum disorder (ASD) is a heterogeneous neurodevelopmental condition involving persistent deficits in social communication and interaction, and restricted, repetitive patterns of behavior, interests or activities (APA, 2013). ASD is often accompanied by a range of psychiatric and medical conditions particularly in adulthood (Croen et al., 2015); however, the needs of the adult ASD population can too frequently be left unmet (Howes et al., 2018; Murphy et al., 2016). Sleep difficulties are a life-long feature, documented from early childhood (Elrod et al., 2016; Fadini et al., 2015; Hodge et al., 2014) and often pinned as one of the most frequent medical complaints in adulthood (Baker & Richdale, 2017; Baker et al., 2019a, b; Goldman et al., 2017; Howes et al., 2018; Ming et al., 2008). Since sleep quality has been argued to be a key predictor of quality of life and employment outcomes (Baker et al., 2019a, b; Lawson et al., 2020) and insufficient sleep is a transdiagnostic feature of psychiatric disturbance across general and clinical populations (Harvey et al., 2011), ascertaining the extent and impact of sleep difficulties in the adult ASD population is important for understanding how best to tailor services to their needs.

### Sleep in ASD

Broadly speaking, sleep can be measured subjectively (i.e., via sleep diaries and questionnaires) and objectively (i.e., actigraphy, polysomnography), with each providing distinct and complementary insights (Hodge et al., 2012). Often seen as preferable to subjective report (which can be prone to bias and error), actigraphy typically monitors movement activity via a wrist-watch device from which information about sleep–wake patterns can be non-invasively inferred. Although polysomnography (involving scalp-based EEG recording) can provide a more detailed picture of the microstructural properties of different sleep stages, it has its limitations that can be overcome by actigraphy (including usability with larger sample sizes, lower cost, less disruption to routine, and easy use within the home environment). Morgan et al (2020) carried out a meta-analytic systematic review of eight datasets examining sleep difficulties in adults with ASD (aged 16 years or older). Regarding the subjective data (n = 286), adults with ASD reported lower subjective sleep quality, lower sleep efficiency, more wake after sleep onset (WASO), longer sleep onset latency (SOL), and more time spent napping compared to adults without ASD, but did not differ on self-reported total sleep time (TST) or rise time. For the objective actigraphy data (n = 393), adults with ASD presented significantly higher sleep fragmentation, time spent in bed, WASO and SOL and significantly lower SE, but there was again no group difference for TST. This pattern of decreased sleep efficiency and frequent night waking in adults with ASD is somewhat consistent with meta-analyses of data from children with ASD (Deliens et al., 2015; Díaz-Román et al., 2017; Elrod & Hood, 2015), with the exception that sleep efficiency appears to be consistently poorer in adults, TST tends to be reduced in children but not in adults, and there tends to be better consistency between subjective and objective measures in adults. However, many of the studies reviewed by Morgan et al. had small samples (i.e., largest n = 40, smallest n = 10). Furthermore, many recruited samples aged 16 years and over, yet significant developmental changes are still influencing sleep architecture at this point in adolescence (Carskadon, 2011), and thus a clear picture of the nature of sleep difficulties in the adult ASD population is lacking. Jovevska et al. (2020) found that individuals with ASD aged 15–80 (n = 297) self-reported poorer sleep quality and longer SOL relative to individuals without ASD; however, when examining specific age bands these group differences were only observed during early-adulthood (20–39 years) and middle age (40–59 years), suggesting these age groups may be particularly worthy of further investigation. Finally, the meta-analysis by Morgan et al. also focussed on sleep variables averaged over a number of days, but some research has suggested that intra-individual variability in these measures may be more important for affective and cognitive functioning (Bei et al., 2017; Dillon et al., 2015; Phillips et al., 2017). Greater night-to-night variability in sleep duration is a clinical feature of insomnia (Sánchez-Ortuño & Edinger, 2012) that impacts upon daily functioning (Carey et al., 2005) and can indicate misalignment of the sleep/wake cycle (Becker et al., 2017). Indeed, a preliminary examination of intra-individual variation in sleep parameters in 14 adults with ASD found more night-to-night variability in SOL, TST, sleep efficiency and sleep fragmentation (as captured by actigraphy) compared to controls (Hare et al., 2006).

### Sleep and Mental Health

There is a well-established relationship between sleep and mental health that is often conceptualised as dynamic and bidirectional (e.g., Fang et al., 2019; Gregory & Sadeh, 2012; Van Dyk et al., 2016). Aligning with this, disruptions in sleep have been shown to be both a precursor to (Roberts et al., 2008) and a consequence of (Dahl & Harvey, 2007) clinical mental health symptoms. The mechanisms that underlie this complex relationship are not well understood, although it has been suggested that top-down inhibitory networks can be disrupted under conditions of sleep deprivation, impairing emotional regulation, hampering the control of intrusive thoughts, and worsening sleep itself (Harrington & Cairney, 2021). It has also been claimed that sleep difficulties might exert a greater impact on mental health in the ASD (than the neurotypical) population, as a consequence of sleep difficulties exacerbating existing affective problems (Malow et al., 2006; Schreck et al., 2004). Broadly supporting this, 26–57% of individuals with ASD report anxiety or depression in their lifetimes (Wigham et al., 2017) and subjective sleep report measures generally correlate with anxiety and/or depression symptoms and measures of psychological wellbeing in children and adolescents with ASD (Allik et al., 2006; Delahaye et al., 2014; Jovevska et al., 2020; Uren et al., 2019). However, actigraphy studies of developing ASD populations have produced more inconsistent results (Fletcher et al., 2017), with only some studies reporting associations between SOL and depression (Richdale et al., 2014) and between variability in sleep efficiency and anxiety (Bangerter et al., 2020). Very few studies have examined these associations in adults. Baker et al. ( 2019a, b) ran correlations between symptoms of anxiety and depression and sleep (as measured by 14 nights of actigraphy) in 48 adults with and without ASD (aged 21–44 years). Although trait anxiety and depression were largely not associated with variance in the objective sleep measures, depression symptoms did predict WASO but only for adults with ASD who also had affective problems. This again calls for more research into relationships between affective problems and objectively measured sleep in the adult ASD population.

The somewhat inconsistent support for associations between objective sleep and markers of mental health reported in ASD concords with the broader literature examining these associations outside the ASD population. In general population (GP) samples, whilst variables such as self-reported sleep duration and subjective sleep quality are repeatedly shown to be associated with psychological wellbeing (e.g., Faubel et al., 2009; Lund et al., 2010), findings from objective measures of sleep have been less consistent. For example, Ryff et al. (2004) found that objectively measured sleep duration (captured via eyelid motion monitoring) was positively associated with psychological wellbeing in females aged 65 years and older; however, Jean-Louis et al. (2000) found no associations between actigraphy measures of sleep duration, SOL, and sleep efficiency with wellbeing in an adult GP sample (see also Mezick et al., 2009). As mentioned above, however, it has been argued that objective measures of *intra-individual variability* in sleep duration may be a more powerful marker of wellbeing than averaged measures of sleep duration and efficiency (Lemola et al., 2013).

### Sleep and Cognitive Abilities

Sleep is also directly and actively involved in supporting homeostatic processes that are required for daily cognition (Deak & Stickgold, 2010). There is a general consensus that sleep is an important predictor of attention, working memory, and executive function, and also actively supports the ability to learn and consolidate new material (e.g., Alhola et al., 2007; Lim & Dinges, 2010; Rasch & Born, 2013; Wild et al., 2018). Wild et al. (2018) examined relationships between 12 measures of cognitive ability and self-reported sleep duration in a global sample of > 10,000 adults of varying age. Somewhat surprisingly, adults who slept less than 7 or more than 8 h (~ 50% of the sample) showed reduced cognitive abilities on some, but not all, measures. Specifically, this pattern was strongest for “higher-level” measures of grammatical reasoning and a digit span task capturing verbal working memory, but was not observed for “lower-level” measures of spatial short-term memory.

Only a limited number of studies have addressed relationships between daily cognitive abilities and sleep in the ASD population. Whilst some studies have found associations between parent reported sleep difficulties and measures of verbal and nonverbal IQ in children with ASD (Malow et al., 2009; Taylor et al., 2012; Veatch et al., 2017) others report weak or absent correlations (Krakowiak et al., 2008; Mayes & Calhoun, 2009), and only weak and preliminary evidence is available for adults (Limoges et al., 2013). Therefore, there is again a lack of data on the presence and nature of a broad relationship between cognitive performance and sleep in adults with ASD, and associations in the GP are not ubiquitous.

### The Present Study

The above literature points to a lack of understanding of the nature of sleep disruption in the adult ASD population, and crucially, the extent to which sleep is associated with affective and cognitive performance in this population. To address this, we carried out a pre-registered secondary data analysis of UK Biobank data. The UK Biobank database comprises data from > 502,000 UK residents (aged 37–73 years at recruitment) who were all asked to report their average sleep duration, and a subset of whom (> 91,000) had their daytime activity (and sleep) recorded via wrist-watch accelerometer for up to 7 days. Using this database, we examined the nature of the associations between sleep and affective variables (psychological wellbeing and lifetime experience of poor mental health) and cognitive variables (verbal short term memory, numerical reasoning, and educational attainment) in adults with and without ASD. A number of studies have already published findings utilising aspects of these data. For example, Lyall et al. (2018) used Biobank accelerometer data from 91,105 participants to derive a circadian relative amplitude variable as a measure of circadian rhythm disruption. Lyall et al. found that a lower circadian relative amplitude was associated with an increased risk of depressive disorder, bipolar disorder, mood instability, neuroticism, subjective loneliness, lower happiness, lower health satisfaction and slower reaction times (see also Ferguson et al., 2018, who report associations between mood instability, major depressive disorder, neuroticism and polygenic risk scores for reduced rest-activity cycles in the Biobank population). However, to our knowledge, studies are yet to use this database to compare sleep characteristics in adults with and without ASD.

This study addressed three research questions: (1) Are sleep difficulties (as captured by subjectively reported sleep duration and objectively measured actigraphy) more characteristic of adults with ASD relative to adults without ASD? (2) Is variance in objective sleep (i.e., measured by actigraphy-derived sleep duration, sleep efficiency, night-time activity, night-to-night variability in sleep duration) associated with psychological wellbeing and/or experience of mental distress in adults with and without ASD? (3) Is variance in objective sleep associated with cognitive function (i.e., verbal short term memory and speed of processing) and educational outcomes in adults with and without ASD? We focused on averaged sleep duration (time spent asleep), efficiency (% time asleep from sleep onset to offset) and night time activity (level of activity during sleeping hours) as these variables are comparable to those used in previous actigraphy studies, and night-to-night variability in sleep duration as a proxy for fluctuations in sleep patterns. Our aim was to examine the nature and associates of these continuous sleep characteristics, as opposed to considering the presence or absence of sleep disorder.

Since the adults with ASD self-reported their diagnoses and volunteered and participated independently, they likely experienced fewer symptoms that would interfere with or preclude participation (e.g., communication difficulties, sensory sensitivities) and therefore may not be representative of all adults with ASD. The adults without ASD comprised a GP sample, serving as a valuable reference point to assess the severity of sleep problems in the ASD population. We use the term “general population” to reflect that additional diagnoses were free to vary in both groups, increasing representativeness and revealing the prevalence and nature of sleep difficulties in a naturally occurring sample. The use of a control sample also allowed for the controlling of key confounding variables. For example, lower socioeconomic status has been associated with longer SOL, lower SE, more WASO and poorer self-reported sleep quality in adults (Friedman et al., 2007; Mezick et al., 2008). The other confounding variables included here were, alcohol consumption, which has been found to cause sleep disruption particularly in the second part of the night (Ebrahim et al., 2013); body mass index (BMI) which has been associated with short sleep duration (Taheri et al., 2004); and smoking, which has been linked to longer SOL (Zhang et al., 2006).

We tested the following pre-registered hypotheses (https://osf.io/cvt6j/):

#### H1

ASD status will account for significant variance in subjectively reported hours of nocturnal sleep, with adults with ASD reporting significantly lower sleep duration than adults without ASD^[1]^.

#### H2

ASD status will account for significant variance in objective measures of sleep (i.e., sleep duration, sleep efficiency, night activity, night-to-night variability of sleep duration), with adults with ASD showing reduced sleep duration, reduced sleep efficiency, increased night activity and increased night-to-night variability than compared to adults without ASD.

#### H3

Variance in objective sleep (i.e., sleep duration, sleep efficiency, night activity, night-to-night variability) will be bidirectionally associated with measures of psychological wellbeing and experience of mental distress with ASD status accounting for additional variance.

#### H4

Variance in objective sleep (i.e., sleep duration, sleep efficiency, night activity, night-to-night variability) will be bidirectionally associated with cognitive ability (verbal short term memory and speed of processing) and educational outcomes, with ASD status accounting for additional variance.

## Method

### Participants

The present study utilised data from the UK Biobank, a large prospective study of ~ 502,000 UK residents aged 37–73 years recruited from 2006 to 2010, which has collected extensive lifestyle, demographic, health, mood, cognitive and physical assessments and questionnaire responses, phenotypic and genotypic details about its participants, with ongoing longitudinal follow-up of a wide range of health-related outcomes (Sudlow et al., 2015). The present study (UK Biobank Project 43711) was covered by the general ethical approval for UK Biobank studies from the NHS National Research Ethics Service on 17th June 2011 (Ref 11/NW/0382). Informed consent was obtained for all participants and all data was anonymised. Ethical approval for this secondary data analysis study was also granted by the Department of Psychology Ethics Committee at the University of York.

To address H1, participants were selected from the UK Biobank database if they had completed the subjective sleep duration question (described below) at the baseline assessment (2006–2010). The analysed sample comprised 223 adults with ASD and 501,439 adults without ASD. Questions about diagnoses (including ASD) were included in the baseline assessment; thus, ASD status was captured via self-reported diagnosis. Participants were asked “Have you been diagnosed with one or more of the following mental health problems by a professional, even if you don't have it currently? (Tick all that apply^**[2]**^)”. Participants were classified as having ASD if they ticked “Autism, Asperger's or autistic spectrum disorder” (and were included regardless of other diagnoses), and were eligible for inclusion in the GP group if they did not tick this category (again, regardless of other diagnoses). Approximately 9% of the GP group reported at least one diagnosis, whereas 75% of the ASD sample reported at least one diagnosis in addition to ASD. For both groups, depression (62% ASD; 7% GP) and anxiety (44% ASD; 4% GP) were the most frequently reported diagnoses. As would be expected, the ASD participants also reported markedly more social anxiety and panic attacks than the GP group (66% ASD; 11% GP). See Supplementary Table 1 for full details of additional diagnoses for this sample. Participants were then matched on a ratio of 1:10 (ASD:without ASD) on age, sex, body-mass index (BMI), alcohol intake (never, occasionally, regularly, daily), smoking status (never, previous, current), ethnic origin and Townsend social deprivation scores (derived from postcode of residence, with negative scores reflecting greater affluence) using the MatchIt package in R (Ho et al., 2011). This left a total sample of 220 adults with ASD and 2200 adults without ASD. Including a larger control sample when looking at neurodevelopmental disorders is recommended practice (Fombonne, 2016). All background variables were collected as part of the baseline assessment (2006–2010) via a touchscreen lifestyle questionnaire. We also accessed employment information for all participants, which we transformed to in/out of paid employment (see Table 1).

**Table 1:** Descriptive Statistics for background variables and sleep measures

	ASD (n = 220)	Min–max	GP (n = 2200)	Min–max	Group differences (p values)
Female	N = 66 (30%)	–	N = 662 (30.1%)	–	–
In employment	63.18%	–	70.04%	–	.04
Age	59.34 (7.76)	45–75	59.08 (8.26)	45–78	.65
BMI	27.19 (5.15)	18.1–44.3	27.22 (4.43)	16.6–50.1	.94
Alcohol intake		–		–	.98
Daily or almost daily (%)	17.7		18.5		
Three/four times a week (%)	20.5		21.3		
Once/twice a week (%)	16.4		16.5		
One to three times a month (%)	15.9		16.7		
Special occasions only (%)	13.6		12.2		
Never (%)	15.9		14.8		
Ethnicity		–		–	.96
White/British/Irish (%)	95		94.6		
Black (Caribbean) (%)	0.5		0.9		
Asian (Chinese/Indian) (%)	1.8		2.0		
Mixed race (%)	0.5		0.6		
Other (%)	2.3		1.9		
Smoking		–		–	.88
Current (%)	11.4		11.4		
Never (%)	61.4		69.8		
Previous (%)	27.3		28.77		
Subjective sleep (n h/night)	7.28 (1.42)	4–16	7.11 (1.17)	3–20	.096

	N = 83		N = 824		
Objective sleep					
Sleep duration in minutes (5 day average)	419.97 (67.62)	154.57–584.67	429.09 (59.93)	27–623.5	.24
Sleep efficiency (5 day average)	0.72 (.09)	.30–.93	0.75 (.08)	.28–1.0	.03
Night activity (5 day average)	9.53 (4.43)	3.35–31.07	10.02 (6.16)	2.43–76.14	.36
Night-to-night duration variability	16.75 (19.55)	3.27–121.35	13.46 (13.63)	.14–258.99	.13
Cognitive ability					
Speed of processing	20.26 (5.88)	2–32	21.11 (5.30)	0–38	.28
Verbal short-term memory	7.21 (1.59)	2–11	7.08 (1.37)	2–11	.52
Educational attainment					
College/university degree or higher	54.10%	–	46.97%	–	.30
In employment	68.85%	–	76.03%	–	.22
Wellbeing and mental health					
Wellbeing^+^—generally happiness	3.77 (1.11)	1–6	4.51 (.80)	1–6	< .001
Wellbeing^+^—happiness about health	3.73 (1.26)	1–6	4.32 (1.01)	1–6	< .001
Wellbeing^+^—life is meaningful	2.95 (1.10)	1–5	3.60 (.96)	1–5	< .001
Positive history of lifetime MH illness	79.0%	–	41.0%	–	< .001

Ns for subjective sleep 218 for ASD, 2138 for GP

^+^Higher number = higher wellbeing

To address H2, participants were selected from the UK Biobank Database if they had contributed accelerometer (actigraphy) data: 88 of the above participants with ASD and 103,604 without ASD (collected between 2013 and 2015). Participants were removed if their accelerometer data could not be calibrated (i.e., using a data field provided by UK Biobank) or if their wear time was deemed by UK Biobank as not being sufficiently long to get a stable measure of their physical activity (leaving n = 84 with ASD and n = 96,610 without ASD) (these parameters are described in detail by Doherty et al., 2017). Participants were then matched on a ratio of 1:10 (ASD:without ASD) on the same list of background variables as detailed above. A further participant with ASD was lost at this stage, due to missing alcohol intake and ethnicity data, thus leaving n = 83 in the ASD group.

Testing of H3 and H4 included all participants with usable accelerometer described above (collected between 2013 and 2015) who had available data from the wellbeing, mental distress (collected as part of an online mental health questionnaire in 2016–2017), cognitive (numeric memory and digit symbol substitution, collected as part of the online follow up study 2014–2015) and educational qualifications variables (collected at the baseline assessment 2006–2010). The sample size for the ASD groups for H1–4 was thus opportunistic; matching each participant with ASD to 10 participants without ASD was implemented to achieve greater statistical power to detect differences associated with ASD status. More specifically, a larger control group reduces variability in that group, thus increasing the power to detect differences in comparison to the target group, in this case ASD, which is harder to recruit.

### Measures

All sleep measures are defined in Table 2.

**Table 2:** The sleep measures used in this study

Sleep measure	Definition
Subjective sleep duration	Self-reported response to “About how many hours of sleep do you get in every 24 h? (please include naps”
*Accelerometer measures*	
Sleep duration	The sum of periods of at least five minutes with no change larger than 5° associated with the z axis of the accelerometer (in minutes)
Sleep efficiency	Percent time asleep, calculated as sleep duration (see above) divided by the time elapsed between the start of the first sleep bout and the end of the last sleep bout
Night activity	Activity during night hours (in milligravity units)
Night to night variability in sleep duration	(Standard deviation of sleep duration across the nights of measurement/average sleep duration across the nights of measurement) * 100

#### Subjective Sleep

During the baseline assessment participants were asked to self-report their average daily sleep duration in hours and minutes. Specifically, they were asked “About how many hours sleep do you get in every 24 h? (Please include naps)”. If the participant activated the Help button they were shown the message: “If the time you spend sleeping varies a lot, give the average time for a 24 h day in the last 4 weeks.”

#### Objective Sleep

Participants were invited to wear an Axivity AX3 wrist-worn accelerometer (Axivity, Newcastle upon Tyne, UK) on their dominant wrist for up to seven days, continuing with their normal day-to-day activities (for more procedural info see Doherty et al., 2017). Data pre-processing was performed by the UK Biobank accelerometer expert working group. Following Jones et al. (2019), the open source R package GGIR was used to infer accelerometer non-wear and extract the “z angle” across five second epochs from the time-series data for subsequent use in estimating the sleep period time window and sleep episodes within it. The “z angle” is calculated from three orthogonally positioned acceleration sensors that use gravitational (g) units (1 g = 1000 milligravity units) to determine the position of the arm. From this, we extracted nighttime sleep duration (in minutes, also referred to as “sleep period time” or SPT, defined as the sum of periods of at least 5 min with no change larger than 5° associated with the z-axis of the accelerometer, see van Hees et al., 2013, for further description), sleep efficiency (% time asleep within the SPT window; calculated as sleep duration divided by the time elapsed between the start of the first inactivity bout and the end of the last inactivity bout, which equals the SPT window duration) and night activity data (activity during night hours in milligravity units) for each participant. Night to night variability in sleep duration was calculated as follows: (standard deviation of sleep duration across the 5 nights of measurement/the average sleep duration across the 5 nights of measurement) * 100 and log transformed for path analyses (see Lemola et al., 2013; Rowe et al., 2008). When considering the whole sample, the majority of participants, n = 753/907, provided 5 nights of actigraphy data, with n = 89 providing 4 nights and n = 65 providing 3 nights. On the basis of data simulations, Doherty et al. (2017) recommends the use of a minimum of 3 nights.

#### Psychological Wellbeing

A subset of UK Biobank participants were invited to complete an Online Mental Health Questionnaire (MHQ “Thoughts and Feelings”) designed to assess lifetime symptoms of mental disorders. Participants’ responses from three questionnaire items relating to wellbeing were included to capture positive emotion (“In general how happy are you?” and “In general how happy are you with your health?”; 1 = extremely happy; 6 = extremely unhappy) and eudemonic wellbeing (“To what extent do you feel your life to be meaningful?”; 1 = not at all, 5 = an extreme amount). A further question relating to lifetime experience of mental distress was also included in the analyses (“In your life, have you suffered from a period of mental distress that prevented you from doing your usual activities?”; yes 1, no 2). All of these questions also had “do not know” and “prefer not to answer” options that were classed as missing data.

#### Cognitive Function

Two cognitive measures were used from UK Biobank (both administered on-line): digit symbol substitution and numeric memory. *Digit symbol substitution* provided a measure of speed of processing. Participants were presented with a series of grids in which symbols were to be matched to numbers according to a key presented on the screen. The score was the number of correctly completed grids in 2 min. The instructions given to participants were as follows: “This is a code-breaking game. A code is given at the top of the page linking a symbol to a number. In the bar at the bottom of the page, place the correct number in the box under each symbol according to the code. Working from left to right select the correct number using the number pad on the screen. Please work as quickly and accurately as you can. You will have two minutes to do as many as you can”. *Numeric memory* was assessed via a digit span task and provided a measure of verbal short term memory. The participant was shown a 2-digit number, displayed for 2000 ms + (the number of digits [i.e., 2] × 500 ms), followed by a wait period of 3000 ms, after which the participant was asked to enter the digits via a keyboard. The number became one digit longer each time they remembered correctly (up to a maximum of 12 digits). The digits were randomly ordered on each presentation, and no displayed digit was the same as the previous (or previous but one) digit. The test ended upon five successive incorrect attempts for two or fewer digits, and two successive incorrect attempts for three or more digits. The score used for each participant was the maximum number of digits remembered correctly (up to 12). The instructions given to participants were: “In the next game you will be shown a number to remember. The number will then disappear and after a short while you will be asked to enter it into the number pad on the screen. The number will become longer each time you remember correctly. Press ‘Next’ for a short video demonstration.”

#### Educational Attainment

At the baseline Biobank assessment participants were asked to indicate whether they had obtained GCSEs or equivalent, A levels or equivalent, a college or University degree or a professional qualification (e.g., nursing, teaching) or none of the above. We transformed this into a binary variable according to whether participants did or did not have a college or university level degree.

### Statistical Analysis

*H1* anticipating a moderately skewed distribution and unequal variance across the groups, we utilised linear regression with robust estimation, which relaxes the parametric assumptions associated with linear regression, with the outcome variable subjective hours of nocturnal sleep and the categorical predictor ASD status. We also examined the effect of sex on these associations.

*H2* the objective sleep measures were weakly correlated and did not load onto a single factor. Linear regression with robust estimation was therefore used with each sleep variable in turn (sleep duration, sleep efficiency, night activity, night-to-night variability of sleep duration) and ASD status as a categorical predictor. We used the 5-day averaged variables for the analyses of sleep duration, sleep efficiency and night activity, and checked our findings against the individual day data using mixed linear regression models. To clarify, this modelled day as a fixed factor and participant ID as a random factor to account for the non-independence of the data from each individual across multiple days. We also examined the effect of sex on these associations.

*H3/4* we investigated the inter-relationships between objective sleep measures (sleep duration, night activity, and sleep efficiency variables averaged across 5 days, and night-to-night variability in sleep duration), cognitive ability, educational attainment and psychological wellbeing within a path analysis framework in individuals with and without ASD, conducted within MPlus. Using the previous literature as our guide, correlated models (anticipating bidirectional relationships between sleep and variables of interest) were evaluated.^1^ Our first stage of evaluation was to remove all non-significant associations. We then evaluated, using interaction terms, if and how ASD status influenced the nature of these relationships. We calculated the interaction term within the model then tested its significance. If significant, we evaluated the models within each group separately using a grouping function within MPlus, allowing us to model the ASD and GP groups separately within the same overall model. All models were saturated, so one non-significant path (with the highest p value) was removed to create an unsaturated model with interpretable model fit statistics. As per our pre-registration, the *p* value of significance is corrected to .01 due to the large sample size and the multiple comparisons made. Any results between the traditional value of .05 and .01 are treated as marginal.

## Results

The data analysed here is available subject to the usual UK Biobank access procedures and thus cannot be made openly available (www.ukbiobank.ac.uk). Descriptive statistics for the subjective sleep, objective sleep measurements, cognitive ability, educational, wellbeing outcomes and other background variables (sex, employment status) for each group (ASD and GP) are provided in Table 1.

### Group Comparisons.

#### Subjective Sleep Duration (H1)

There was no effect of ASD status on subjective sleep duration [B = 0.17, 95% CI (− 0.03, 0.36), β = .04; *p* = .096], nor was there an interaction between sex and ASD status. Given that the ASD sample utilized here had relatively high employment rates (see Table 1), and unemployment has been linked to sleep difficulties and could therefore account for the lack of an effect of ASD status on subjective sleep duration, an additional exploratory analysis examined the role of employment in predicting subjective sleep duration. We found that those who were unemployed perceived that they slept *longer* (M = 7.41, SD = 1.59) than those in employment [M = 7.07, SD = 0.96; B =  − 0.40, 95% CI (− .53, − .28), β =  − .16, *p* < .001] and there was no interactive effect with ASD status (*p* = .18). This suggests that the higher than expected rates of employment in the ASD group are unlikely to be masking group differences in subjective sleep duration.

#### Objective Sleep Parameters (H2)

There was no group difference for sleep duration [B =  − 9.12, 95% CI (− 24.18, 5.94), β =  − .04, *p* = .24], night activity [B =  − 0.49, 95% CI (− 1.53, 0.55), β =  − .02, *p* = .36] or night-to-night variability in sleep duration [B = 9.83, 95% CI (− 3.24, 22.90), β = 0.07, *p* = .14]. There was no interaction between sex and ASD status in the prediction of sleep duration, night activity or variability in night-to-night sleep duration.

There was a marginally significant difference in sleep efficiency, with higher sleep efficiency in the GP group than the ASD group [B =  − 0.02, 95% CI (− 0.04, − 0.002), β =  − .08, *p* < .05]. There was also a marginally significant interaction with sex [B = 0.06, 95% CI (0.01, 0.10), β = .16, *p* < .05]. Whilst females without ASD showed higher sleep efficiency than males without ASD [M = .77, SD = .08 for female GP; M = .73, SD = .08 for male GP; B =  − 0.04, 95% CI (− 0.05, − 0.03), β =  − .23, *p* < .001], males and females with ASD showed no difference in sleep efficiency [M = .71, SD = .12 for female ASD; M = .73, SD = .08 for male ASD; B = 0.02, 95% CI (− 0.03, 0.07), β = .09, *p* = .45]. There was also significantly reduced sleep efficiency in females with ASD compared to females without ASD [B =  − 0.06, 95% CI (− 0.10, − 0.02), β =  − .19, *p* < .01] whereas there was no difference in sleep efficiency in males with and without ASD [β =  − 0.001, 95% CI (− 0.02, 0.02), β =  − .005, *p* = .90].

The above results used the 5-day average variables. We conducted a validation analysis using the individual data from each day within a mixed effects regression with participant ID as the random factor, controlling for the non-independence of the available datapoints from each individual. Day was entered as a fixed effect factor. Additional interaction effects between day and group, sex and day and day, sex, and group were conducted for each outcome variable. The results for sleep duration and night activity were identical to the findings from the average variables with no additional significant interaction or main effects of day. For sleep efficiency, the marginally significant main effect above was statistically weaker but nevertheless comparable [B =  − 0.02, 95% CI (− 0.04, 0.0005), *p* = .056]. The significant gender by group interaction for sleep efficiency was still marginally significant [B = 0.05, 95% CI (0.005, 0.10), *p* < .05] and due to the same effects as reported above. There were no other significant interactions. Therefore, in general, the results from the summary variables were replicated within the mixed effects model which retained the individual day data. The 5-day average variables were used for all subsequent analyses.

### Relationships Between Psychological Wellbeing and Objective Sleep (H3)

Individuals with ASD were less happy overall [B =  − 0.74, 95% CI (− 0.99, − 0.48), β =  − .28, *p* < .001], less happy with their health [B =  − 0.60, 95% CI (− 0.89, − 0.31), β =  − .19, *p* < .001], and felt life had lower meaning [B =  − 0.65, 95% CI (− 0.90, − 0.40), β =  − .22 *p* < .001]. They were also over five times more likely to have experienced a lifetime mental health illness [OR = 5.41, 95% CI (3.09, 9.49), *p* < .001]. None of these associations were influenced by sex.

We examined relationships between psychological wellbeing (general happiness, health happiness and “life is meaningful” questions) and sleep. The first two questions were measured on a six-point Likert scale, from 1 to 6, and the “life is meaningful” question was measured on a five-point Likert scale, from 1 to 5. We treated these scales as an ordinal approximation of a continuous measurement, as all variables had at least five categories and research indicates that these types of Likert data can be treated as continuous, even with extremely skewed distribution (Norman, 2010). As the variables were not normally distributed (though not heavily skewed), we used the MLR estimator. To assess the appropriateness of treated the measures as continuous variables, we also replicated the final results using a binary version of the variables (unhappy/happy dichotomy for general happiness and health happiness and not at all/a little vs. a moderate amount/very much/extreme amount for the meaningful life variable). This sensitivity analysis produced equivalent results, with the exception of the links between sleep duration and general happiness and health happiness, which became non-significant.

**Fig. 1:**
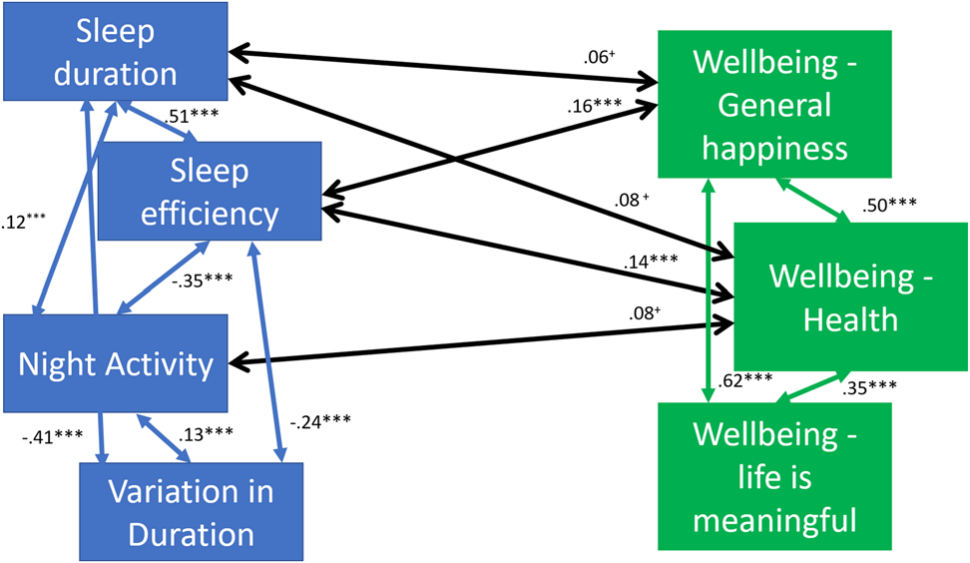
Inter-relations between sleep and psychological wellbeing in the reduced model. *Note* ^+^*p* < .05, *p < .01, **p < .005, ***p < .001

As shown in Fig. 1, there were associations between psychological wellbeing and the objective sleep measures. The final model had excellent fit to the data (Chi Square p value = .61, RMSEA < .001, CFI = 1.0, TLI = 1.0, SRMR = .02). The associations were most prominent with sleep efficiency, with both general happiness and health happiness associated with better sleep efficiency, indicating that as wellbeing reports of happiness worsen, sleep efficiency also gets worse. There was also a marginal association between having lower health happiness and more night activity. There were no associations between the sleep variables and whether participants thought life was meaningful.

**Fig. 2:**
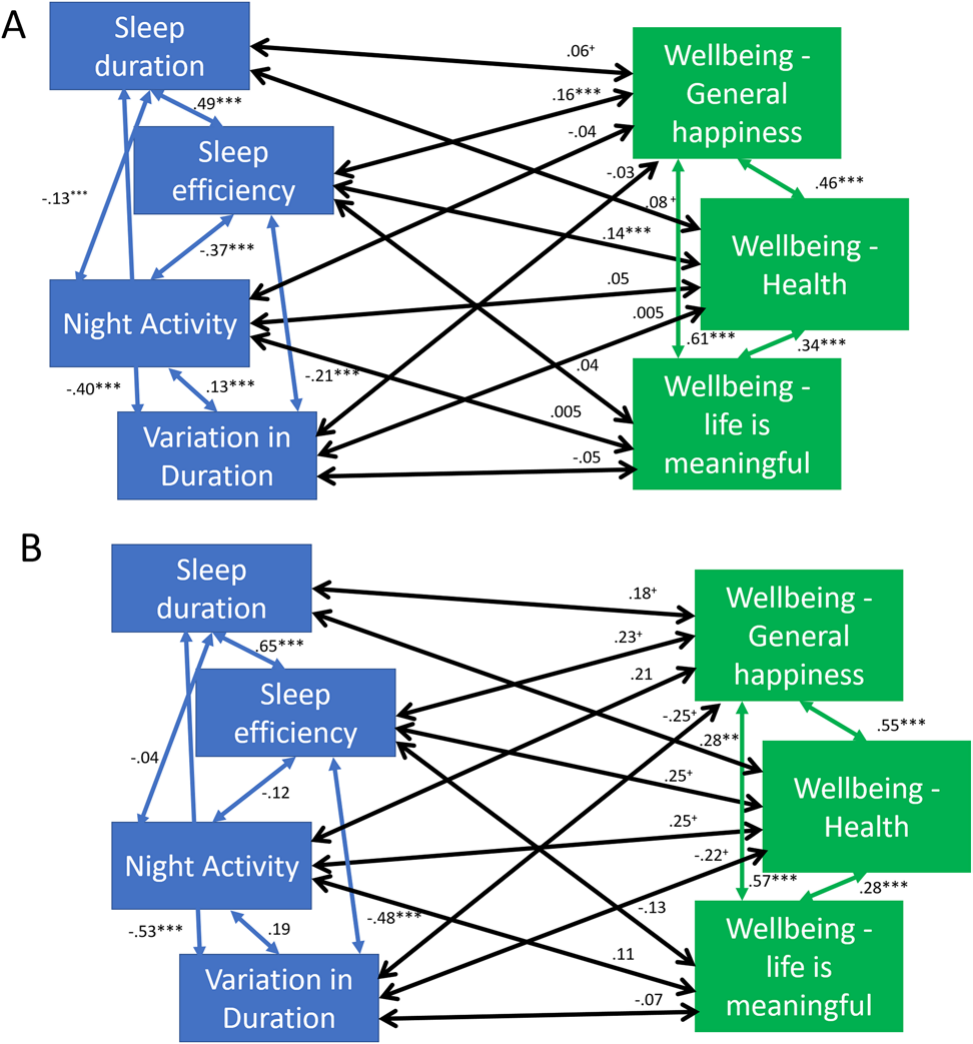
**A** The full wellbeing correlation model for the GP group (N = 824). **B** The full wellbeing correlation model for the ASD group (N = 83). *Note* ^+^*p* < .05, *p < .01, **p < .005, ***p < .001

We then evaluated the interactive ASD group effects on the model within the full model where psychological wellbeing correlated with sleep measurements. We found highly significant interactions between group and each sleep variable in the correlations with general happiness, health happiness and the life is meaningful variable. Two models were run (for each group) to evaluate how the effects differed between groups. There were much stronger standardised coefficients in the ASD group than in the GP group (see Fig. 2A, B). Although, due to the lower power in the ASD group, many of the effects became marginally significant, on occasion the significance did increase in the ASD group.

### Relationships Between Lifetime Experience of Mental Distress and Objective Sleep (H3)

**Fig. 3:**
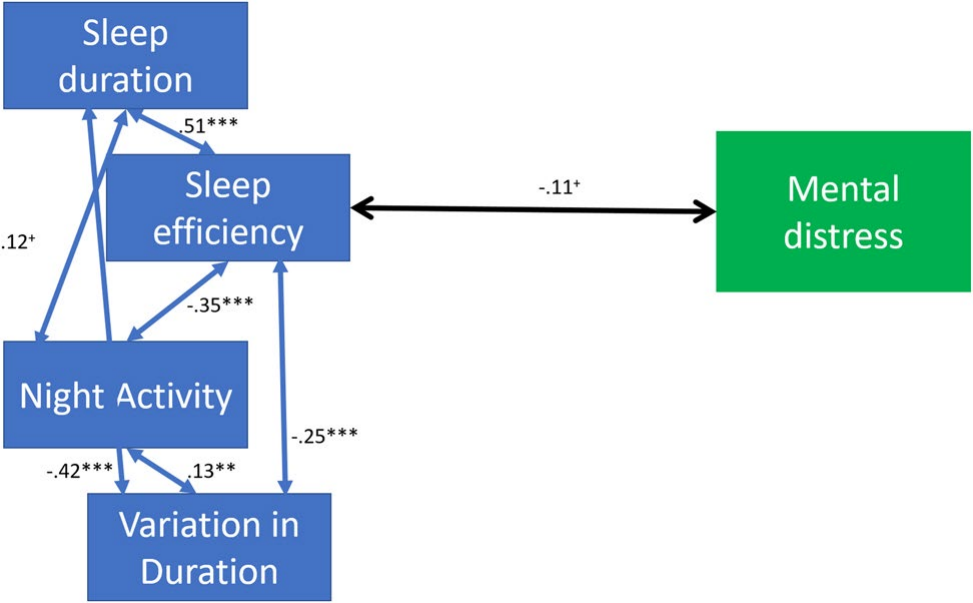
Inter-relations between sleep and mental distress in the reduced model. *Note* ^+^*p* < .05, *p < .01, **p < .005, ***p < .001

The final model is presented in Fig. 3. The links between sleep duration, night activity and variance in sleep duration were not correlated with lifetime mental illness and were dropped from the model. The final model had excellent fit to the data (Chi Square p value = .16, RMSEA = .028, CFI = .998, TLI = .994, SRMR = .026). Of interest, reduced sleep efficiency was correlated with the presence of lifetime mental health illness/distress (at *p* < .05).

**Fig. 4:**
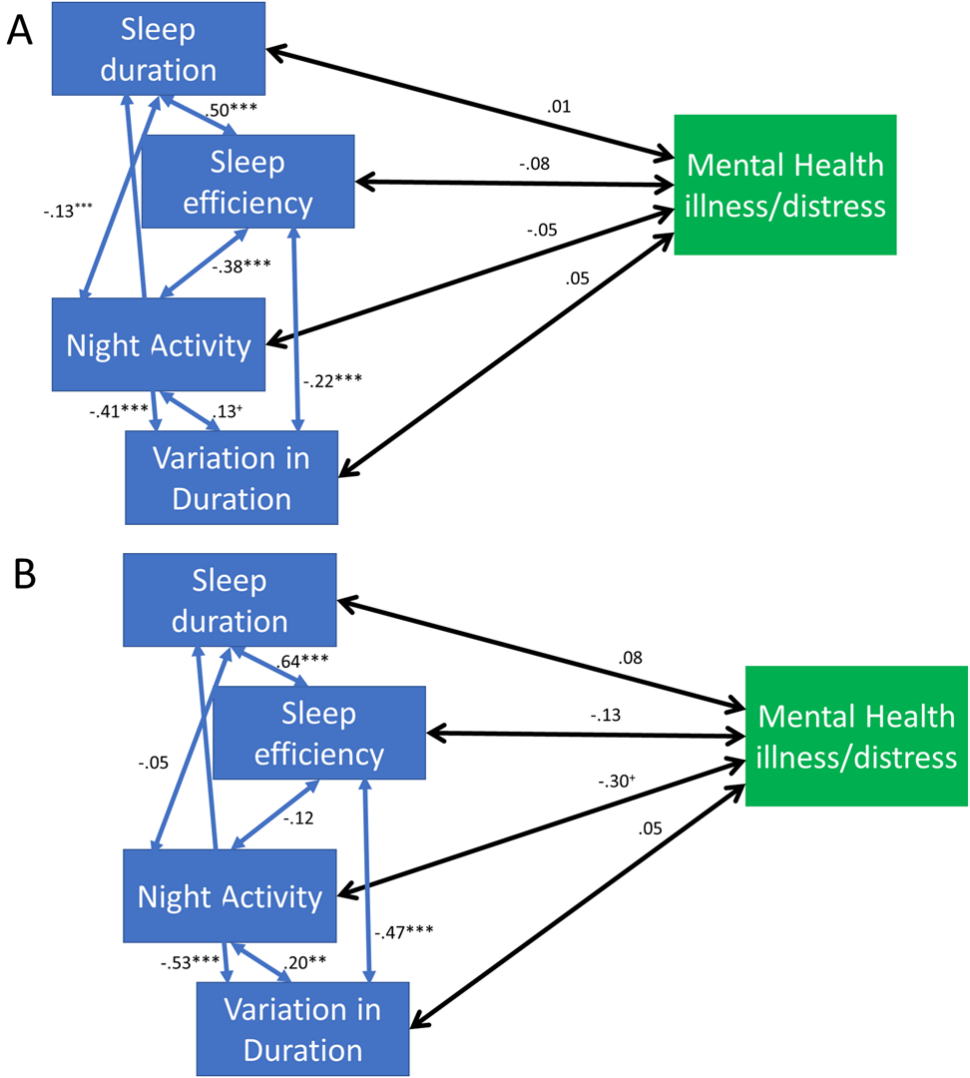
**A** The full mental health illness/distress model for the GP group (N = 824). **B** The full mental health illness/distress model for the ASD group (N = 83). *Note* ^+^*p* < .05, *p < .01, **p < .005, ***p < .001

Upon evaluating group differences in the full model, there were significant interactions between duration (β = .29, *p* < .001), efficiency (β = .28, *p* < 0.001), night activity (β = .21, *p* < .01) and variation in sleep duration (β = .28, *p* < .001) and ASD group in predicting lifetime mental health illness/distress. As with mental wellbeing, the standardised coefficients were stronger in the ASD group when the model was rerun using multigroup functions, excepting variation in sleep duration. This indicated there was a stronger relationship between sleep and lifetime history of mental health illness/distress in the ASD group. However, as can be seen in Fig. 4A, B, only the path between night activity and mental illness/distress reached *p* < .05, likely due to low power in this group.

### Relationships Between Cognitive Ability and Objective Sleep (H4)

There was no effect of ASD status on processing speed [B =  − 0.85, 95% CI (− 2.40, 0.70), β =  − .05, *p* = .28] or verbal short term memory [B = .14, 95% CI (− 0.28, 0.55), β = .03, *p* = .52], and neither of these associations were moderated by sex. We then looked at the inter-relationships between cognitive ability and objective measures of sleep. The full correlated models had a strong absolute fit to the data (Chi Square p value = .97, RMSEA < .001, CFI = 1.0, TLI = 1.0, SRMR =  < .001) but the final model indicated no overall association between the sleep and cognitive variables (see Supplementary Material Fig. 1). The high model fit was likely driven by the strong intercorrelations between the sleep variables. After we deleted the nonsignificant paths sequentially, the pattern of no association between sleep and cognitive variables remained. In order to evaluate whether there were any differences in the paths between cognitive measures and objective sleep measures between the ASD groups, we evaluated the interactive effect between each sleep variable and group in the final model prior to any pruning. There were no significant group interactions in any of the pathways.

### Relationships Between Educational Attainment and Objective Sleep (H4)

We repeated the above analysis looking instead at how educational attainment and objective sleep were associated. First, there was no ASD group difference in educational qualifications [OR = 1.33, 95% CI (− 0.78, 2.27), *p* = .30], nor was this association moderated by sex. Similar to previous results, there was little evidence of an association between educational attainment and objective sleep measures, but there was a marginally significant association between night activity and educational attainment, such that those with college degrees were more likely to have more activity during the night. When we sequentially deleted non-significant pathways, the link between night activity and educational attainment remained marginally significant (β = .09, *p* < .05). There was a good model fit for the final model (Chi Square p value = .75, RMSEA < .001, CFI = 1.0, TLI = 1.0, SRMR = .01). There were no interactive effects with ASD group in the full model (see Supplementary Material Fig. 2).

## Discussion

The present study represents the largest analysis of subjective and objective sleep in adults with ASD to date, addressing the extent to which objective sleep parameters are associated with measures of wellbeing, mental illness, cognitive and educational ability. Importantly, the present UK Biobank ASD sample largely comprised individuals without cognitive impairments who had higher-than-expected employment rates, and it is important to keep this caveat in mind when considering the following key findings. Namely, there was little evidence of group differences in subjectively reported sleep duration or objective accelerometer sleep measures (with the exception of marginally lower sleep efficiency in the ASD group), nor were there associations between sleep and cognitive or educational variables for either group. However, there were clear associations between objective sleep parameters (particularly sleep efficiency) and psychological wellbeing and lifetime experience of poor mental health, and importantly, these pathways were much stronger for adults with ASD than for adults without ASD from the general population.

### Do Adults With and Without ASD Differ on Subjective and Objective Sleep Characteristics?

The mean self-reported daily sleep duration for both groups was ~ 7 h per day, close to the mean 7.2 h of sleep reported per day for the larger UK Biobank population (Dashti et al., 2019), and, counter to predictions, adults with ASD did not report sleeping less than adults without ASD. Furthermore, there were no effects of ASD group status on actigraphy-derived measures of averaged nocturnal sleep duration, night-to-night variability in sleep duration or night activity. Thus, for the present adult ASD sample, who were closely matched to adults from the GP on a range of health-related background variables that have been linked to sleep (i.e., age, sex, BMI, alcohol intake, smoking status, ethnicity, socio-economic status), there was little evidence of differences in subjective and objective markers of sleep. There was, however, a marginal effect of ASD group status on sleep efficiency, with adults with ASD spending proportionally more time awake during their sleep hours than adults without ASD. It should be noted that this 3% group difference in sleep efficiency is small and may not have significant clinical ramifications. Yet, it is of interest that this effect was driven by females: females from the GP sample showed significantly better sleep efficiency than males (counter to previous research e.g., Buysse et al., 1991; Mollayeva et al., 2016; Mong & Cusmano, 2016; Tang et al., 2017), but for the ASD group, females and males did not differ. There was also no group difference in sleep efficiency for males with and without ASD, but females with ASD had poorer sleep efficiency than females without ASD. It is difficult to ascertain whether this effect is carried by the GP females having unusually good sleep efficiency, or whether it is driven by the females with ASD having poorer sleep efficiency than is typical. Nevertheless, since females with ASD are both underrepresented in research and have been argued to be at greater risk of internalising difficulties (see Hull et al., 2020, for a review), this finding may warrant further investigation. It is plausible that an increased incidence of internalizing difficulties in females with ASD puts them at greater risk of sleep difficulties (or vice versa). Indeed, a study of subjective sleep (using the Pittsburgh Sleep Quality Index) compared individuals with and without ASD (n 297 and 233 respectively, aged 15–80 years), and found that self-reported sleep quality was poorer in the ASD group, particularly for females and those with a mental health problem (Jovevska et al., 2020).

Taken together, these results are somewhat consistent with the meta-analysis by Morgan et al (2020). Namely, Morgan et al. also found that TST did not differ in adults with and without ASD, yet they showed more fragmented sleep, longer SOL and poorer sleep efficiency. Thus, despite reduced sleep duration being a feature of childhood ASD (as demonstrated in meta-analyses by Deliens et al., 2015; Díaz-Román et al., 2017; Elrod & Hood, 2015), the present findings add to a growing picture of limited evidence for sleep duration differences in adults with ASD. It is also notable that similar standard deviations and ranges characterised the sleep parameters for each group, thus there was also no evidence of greater within-group variability in the ASD sample. As mentioned, it should be borne in mind that these results only apply to individuals with ASD who also have age-expected cognitive abilities and who are well matched to adults from the GP on a range of health-related background variables. The UK Biobank sample is volunteer-based and is not likely to be representative of the broader UK population (with or without ASD; Fry et al., 2017), and thus, the results should be interpreted with this in mind. The lack of generalisability of the sample is particularly important in the context of sleep, given individuals with ASD who also have intellectual disability and/or greater support needs may experience the most severe sleep disruption and behavioural problems (Cohen et al., 2014; Jovevska et al., 2020) and are thus an important target for future research. It is also important to note that the adults who volunteered for the accelerometer study likely deemed themselves to be tolerant of wearing the device during sleep, and could have biased recruitment away from adults with ASD who had more severe sleep and/or sensory difficulties: This limitation is common to many objective sleep studies of ASD, and calls for the development of sleep monitoring devices that are less invasive (e.g., Chinoy et al., 2021; Li et al., 2021).

One might suspect that the relatively high level of employment in the ASD sample could account for the lack of group differences in sleep quantity. Workforce participation in people with ASD is typically estimated to be 42% on average, compared to 83% in the GP (Hedley et al., 2017); however the present rate of employment was ~ 69% for adults with ASD compared to ~ 78% for adults from the GP. A structured employment schedule facilitates the maintenance of a regular sleeping pattern and unemployment has previously been linked to sleep difficulties in adults with ASD (Baker & Richdale, 2017). However, in an exploratory analysis we found that adults in employment perceived that they slept *less* than those not in employment, and this relationship was similar regardless of ASD status. Therefore, the higher level of employment in the present ASD sample is perhaps an unlikely contender for the absent group differences in sleep quantity.

### Are Relationships Between Psychological Wellbeing, Mental Distress and Objective Sleep Characteristics Stronger for Adults with ASD?

For the sample as a whole, there were very high fit levels for the models examining relationships between objectively measured sleep and psychological wellbeing and lifetime experience of mental distress, consistent with previous research (e.g., Alvaro et al., 2013; Steptoe et al., 2008; Zhai et al., 2018). Notably, associations were most prominent for sleep efficiency, indicating that as sleep efficiency gets worse, wellbeing reports of happiness and chances of experiencing poor mental health or extreme distress worsen. Further to this, there were highly significant interactions between ASD group status and each sleep variable in the correlations with general happiness, health happiness and also the “is life is meaningful” question. These interactions emerged because all pathways were stronger for the ASD group than for the GP group. When running separate models for each group, all standardised coefficients between sleep duration, sleep efficiency and variation in sleep duration and both general happiness and health were higher for the ASD group and retained marginal significance despite the much smaller sample size than in the GP group. Furthermore, the correlation between sleep duration and health happiness retained significance for participants with ASD. Therefore, these data point to a tighter relationship between psychological wellbeing and particularly sleep efficiency in adults with ASD, than in adults in the GP. There were also significant interactions between duration, efficiency, night activity and variation in sleep duration and ASD group status in predicting lifetime mental health illness/distress. That is, the standardized coefficients were again stronger in the ASD group than in the GP group.

These data support previous assertions that the quantity and quality of sleep may exert a greater affective influence in the ASD population than in the GP, potentially owing to sleep exacerbating symptomatology and/or already existing affective problems (Malow et al., 2006; Schreck et al., 2004). Of course, given the correlational nature of the present analyses, it is also possible that poorer mental health has a greater impact on sleep dynamics in this population. Indeed, despite being closely matched to the GP sample on the range of health-related background variables, adults with ASD were less happy overall, less happy with their health, and felt that life had lower meaning. They were also over five times more likely to have experienced poor mental health. These findings closely align with high rates of anxiety and depression previously reported in adults with ASD (Davis et al., 2011; Gray et al., 2012; Lever & Geurts, 2016; Uljarević et al., 2019; Rai et al., 2018; Wigham et al., 2017), particularly in those who have at least age-expected cognitive ability (Sterling et al., 2008). Interestingly, the presence of this stronger association coupled with largely absent group differences in the sleep parameters (with the exception of weak evidence for lower sleep efficiency in the ASD group) suggests a more sensitive relationship between mood and sleep in ASD. Tentatively, this could mean that there is a pressing need to maintain good sleep to perpetuate good mental health and wellbeing in this population.

An important point to clarify is that the stronger associations between sleep efficiency and mental health and wellbeing found in adults with ASD here are not likely to be ASD-specific. It is quite plausible that this tighter relationship also applies to other disorders that are characterized by poor mental health. Relevant to the drive to shift away from treating disorders as discrete categories (e.g., Astle et al., 2021), a valuable future line of research could examine the extent to which this stronger relationship straddles across multiple diagnostic boundaries and represents a transdiagnostic marker of poor mental health (Baglioni et al., 2016).

### Are Relationships Between Cognitive Abilities, Educational Attainment and Objective Sleep Characteristics Stronger for Adults with ASD?

Counter to predictions there were no associations between the sleep measures and cognitive abilities, and ASD status did not moderate these relationships. However, a recent critical review on the relationship between intelligence and sleep concluded that the association between sleep macrostructure and cognitive abilities is only modest at best (Ujma et al., 2020), and thus the smaller sample size used here may account for the lack of significant associations. Furthermore, Wild et al. (2018) who carried out a large (n > 10,000) on-line investigation into self-reported sleep duration and cognitive abilities in adults, found a curvilinear (as opposed to linear) relationship between sleep duration and cognitive abilities. Specifically, adults who consistently slept less than or more than the recommended 7–8 h a night were impaired on tasks of reasoning and verbal abilities relative to adults who slept the recommended amount (including a verbal short-term memory task similar to the one used here). Wild et al. (2018) also found that adults who slept slightly more than their typical amount on the night before cognitive testing performed better, suggesting a more dynamic association between cognition and sleep that was not possible to capture here (i.e., with the objective sleep data collected between 2013 and 2015 and the online cognitive tasks administered in 2016–2017). It should be noted that we did observe a marginally significant association between night activity and educational attainment, such that adults with college level degrees were more likely to be more active during the night. This is consistent with claims that “night owls” are more likely to have higher IQs (Kanazawa & Perina, 2009). However, given the marginal effect, this finding clearly requires replication and further examination.

### Limitations and Conclusions

This research has a number of strengths and thus contributes to addressing an important empirical gap. However, there are a number of limitations that need to be acknowledged when considering the results. For example, beyond concerns over the generalisability of the present sample, the older participants in this study are much more likely to have received ASD diagnoses late or may have been more likely to have been misdiagnosed earlier in life. Both of these possibilities would mean that they would not have received the same level of support that participants who were diagnosed earlier may have received. Therefore, it is possible that sleep may have a lesser impact on psychological wellbeing and life-time experience of mental distress than in populations where better support has been available from a younger age. Another limitation to note is that diagnoses of ASD were only confirmed by self-report and additional information on symptom severity was not available, which may have introduced inaccuracies in group assignment. Furthermore, although the size of the opportunistic ASD sample was the largest used in an adult ASD objective sleep study to date, it is still smaller than desirable. This raises the importance of developing scalable devices for capturing objective sleep parameters that can be used across ethnically, socially and cognitively diverse populations. Finally, although the majority of participants contributed 5 nights of accelerometer data (which has been demonstrated to be sufficient for meaningful analysis by Doherty et al., 2017), a substantial minority contributed 3–4 nights. Ideally, (particularly for estimates of variability in sleep parameters) participants would contribute data over a longer period at multiple time points to provide a richer, more reliable estimate of the sleep characteristics of a given population.

In conclusion, the present study found no strong evidence for globally poorer subjective or objectively measured sleep in adults with ASD relative to carefully matched adults from the GP without ASD, with only weak evidence for poorer sleep efficiency in adults with ASD. However, variability in objective measures of sleep (particularly sleep efficiency) in the adult ASD population was much more strongly associated with key aspects of mental health—namely psychological wellbeing and lifetime experience of mental distress—than in the GP. This exaggerated association between sleep and mental health suggests that adults with ASD may be particularly sensitive to the effects of poor sleep and highlights the need to better understand the causal mechanisms that underpin this relationship. The findings also suggest that sleep efficiency should be considered as part of routine clinical assessment. Even in the absence of clinical-level sleep difficulties, monitoring and optimizing sleep may be important for the prevention and treatment of mental health difficulties in the ASD (and non ASD) population (see Blake & Allen, 2020). Future endeavors to address this question via randomised controlled trials will not only be clinically valuable, but will also offer a route to examining causal pathways. Notably, a number of challenges surrounding psychotherapeutic and psychopharmacological treatment of depression in ASD have been raised (Chandrasekhar & Sikich, 2015), not least, individuals with ASD may be more prone to adverse effects of psychopharmacological treatments for depression, including irritability and sleep disturbance itself (Boyd et al., 2011; Doghramji & Jangro, 2016). Thus, the recognition of the impact and maintenance of good sleep, and support where required, could feasibly improve quality of life in adults with ASD (Mandell, 2018).

## Supplementary Information

Below is the link to the electronic supplementary material.

Supplementary file1 (DOCX 218 kb)

## Footnotes

1 As detailed in our pre-registration we aimed to explicitly compare models where sleep factors predict the variables of interest with models where the variables of interest predict sleep and models assuming a bidirectional relationship (e.g., incorporating correlations rather than unidirectional predictions). We anticipated that we would be able to use comparative model fit statistics (Akaike Information Criteria and Bayesian Information Criteria) to ascertain which model best accounts for the data. However, this proved difficult because the same estimator could not be used for each model, either due to differences in between continuous and categorical predictors or due to convergence issues in MPlus. We were therefore unable to effectively compare the three theoretical models, and in most cases the absolute fit indices were similarly good in all models.
