# Supplementary material for: Stronger Associations Between Sleep and Mental Health in Adults with Autism: A UK Biobank Study
Source: J Autism Dev Disord. 2021 Dec 3;53(4):1543–59. doi: 10.1007/s10803-021-05382-1 (PMC10066094; doi:10.1007/s10803-021-05382-1)

| Diagnosis | GP (N = 2200) | ASD (N = 220) |
| --- | --- | --- |
| No diagnosis | 1994 (90.6%) | -- |
| Social Anxiety/Social Phobia | 11(0.5%) | 66 (30%) |
| Schizophrenia | 2 (0.09%) | 15 (6.8%) |
| Other Psychosis or Psychotic Illness | 6 (0.3%) | 19 (8.6%) |
| Personality Disorder | 2 (0.09%) | 27 (12.3%) |
| Any other Phobia | 6 (0.3%) | 26 (11.8%) |
| Panic Attacks | 36 (1.6%) | 52 (23.6%) |
| OCD | 3 (0.1%) | 31 (14.1%) |
| Mania, Hypermania, Bipolar or Manic-depressive | 8 (0.4%) | 24 (10.9%) |
| Depression | 150 (6.8%) | 137 (62.3%) |
| Bulimia Nervosa | 0 | 14 (6.4%) |
| Psychological Over-eating or Binge Eating | 6 (0.3%) | 23 (10.5%) |
| Autism, Asperger’s or ASD | 0 | 220 (100%) |
| Anxiety or GAP | 82 (3.7%) | 97 (44.1%) |
| Anorexia Nervosa | 6 (0.3%) | 20 (9.1%) |
| Agoraphobia | 2 (0.09%) | 22 (10%) |
| ADD/ADHD | 1 (0.05%) | 19 (8.6%) |

**SM Table 1**. In the GP sample, the majority of people with a diagnosis (N = 206) only had a single diagnosis (N = 133; 64.6%), with 14 having two diagnoses (6.8%), eight four diagnoses (3.9%), three five diagnoses (1.5%), and one person reporting nine diagnoses (0.5%). In the ASD sample, a minority only had a single diagnosis of ASD (N = 55; 25%), with 48 having two diagnoses (21.8%), 40 three diagnoses (18.2%), 23 four diagnoses (10.5%), 19 five diagnoses (8.6%), nine six diagnoses (4.1%), seven seven diagnoses (3.2%), two individuals each having eight and nine diagnoses (0.9%, respectively), three with eleven diagnoses (1.4%) and 1 individual had twelve, fourteen and fifteen diagnoses, respectively (0.5% each).

**SM Figure 1**. Inter-relations between sleep and cognitive measures (Beta coefficients presented; below non-significant pruning). Note: + *p* <.05, **p* <.01, ***p* <.005, ****p* < .001. Dotted line was non-significant and removed to create an unsaturated model.


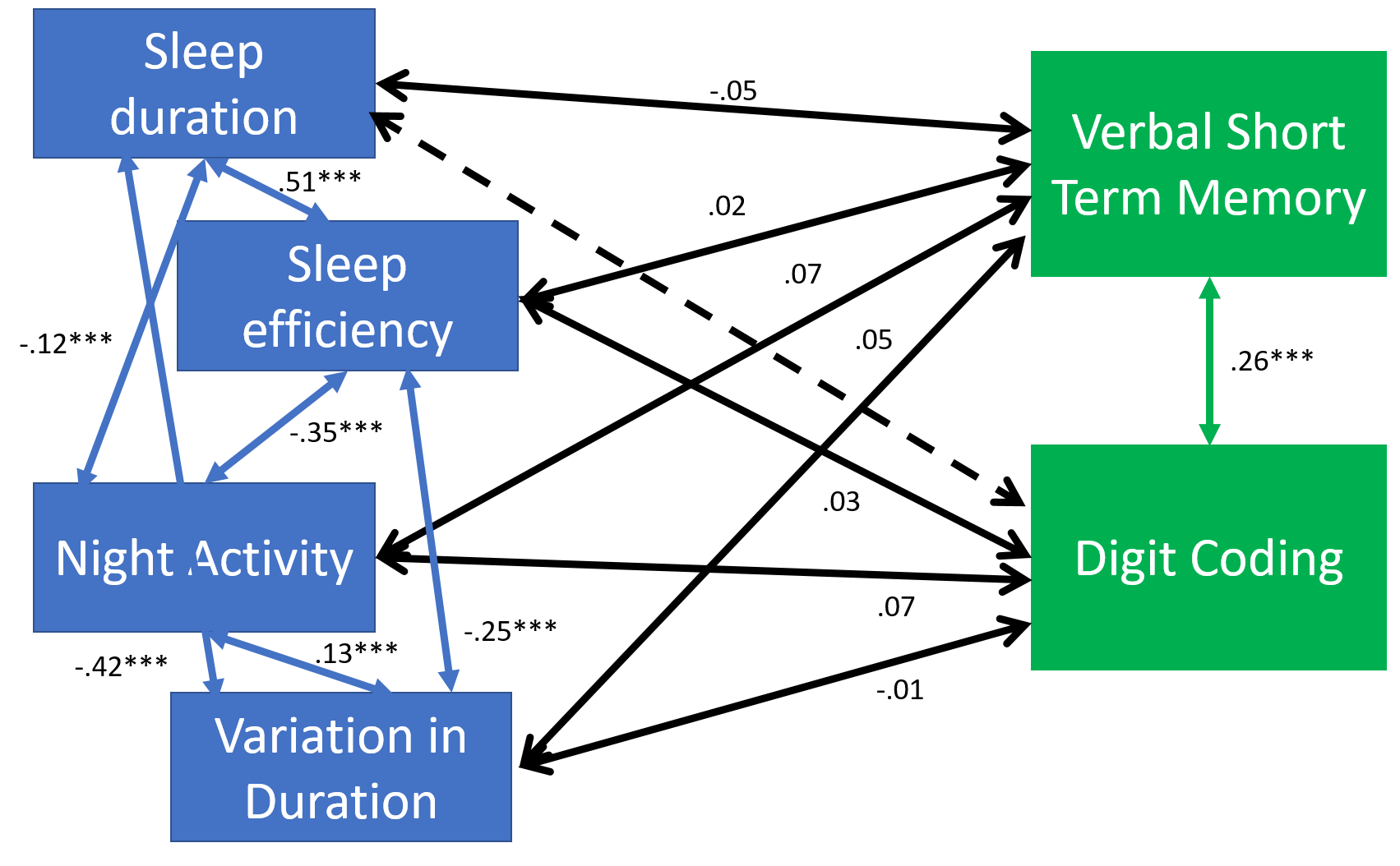


**SM Figure 2**. Inter-relations between sleep and educational attainment. *Note*: + *p* <.05, **p* <.01, ***p* <.005, ****p* < .001. Dotted line was non-significant and removed to create an unsaturated model.


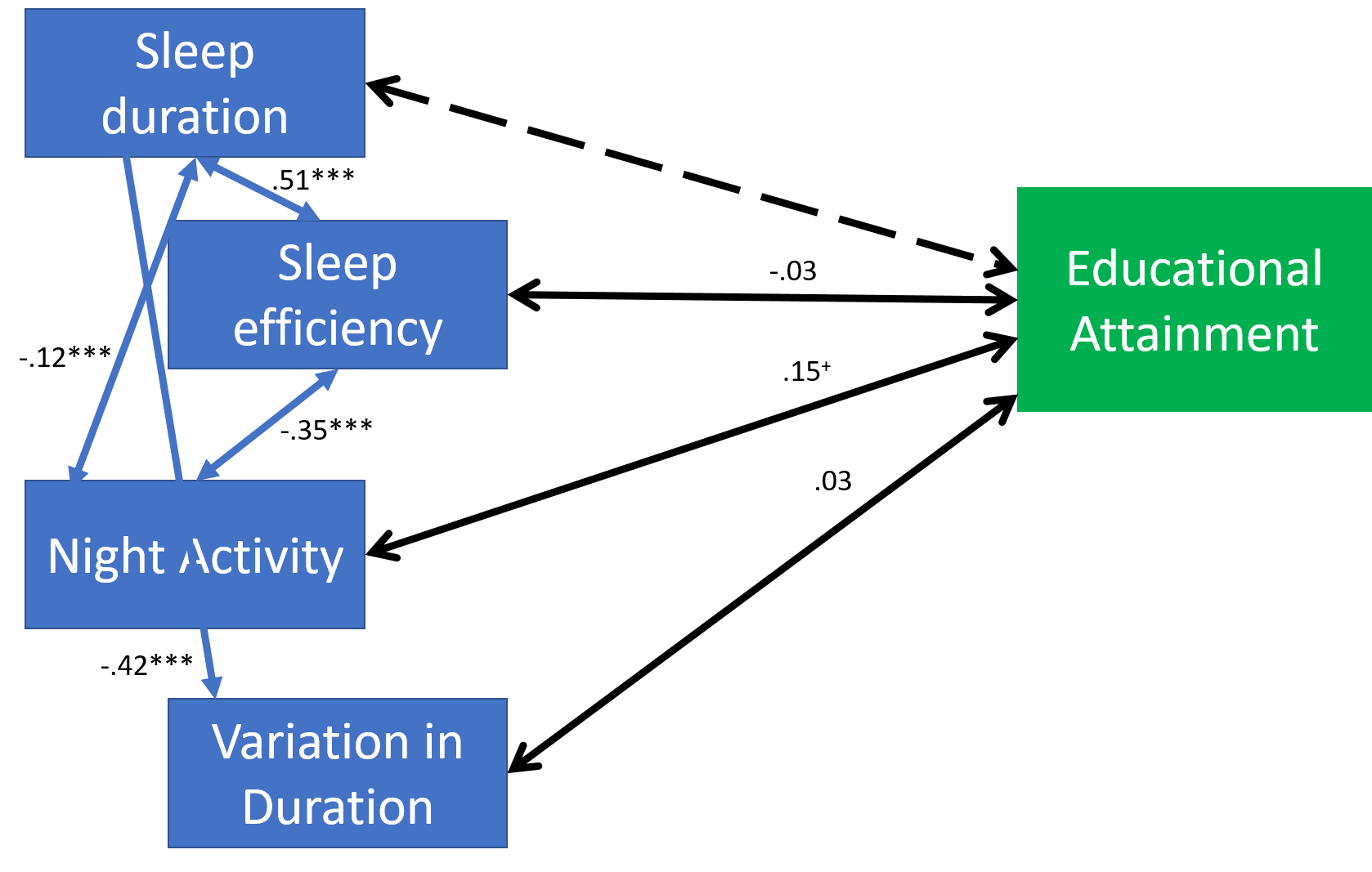

Supplement: Supplementary file 1 — Supplementary file1 (DOCX 218 kb) [file 10803_2021_5382_MOESM1_ESM.docx]
